# Multiple cues produced by a robotic fish modulate aggressive behaviour in Siamese fighting fishes

**DOI:** 10.1038/s41598-017-04840-0

**Published:** 2017-07-05

**Authors:** Donato Romano, Giovanni Benelli, Elisa Donati, Damiano Remorini, Angelo Canale, Cesare Stefanini

**Affiliations:** 1 The BioRobotics Institute, Scuola Superiore Sant’Anna, Viale Rinaldo Piaggio 34, 56025 Pontedera, Pisa, Italy; 2 Department of Agriculture, Food and Environment, University of Pisa, via del Borghetto 80, 56124 Pisa, Italy; 3 Department of Biomedical Engineering and Robotics Institute, Khalifa University, PO Box, 127788 Abu Dhabi, UAE

## Abstract

The use of robotics to establish social interactions between animals and robots, represents an elegant and innovative method to investigate animal behaviour. However, robots are still underused to investigate high complex and flexible behaviours, such as aggression. Here, *Betta splendens* was tested as model system to shed light on the effect of a robotic fish eliciting aggression. We evaluated how multiple signal systems, including a light stimulus, affect aggressive responses in *B*. *splendens*. Furthermore, we conducted experiments to estimate if aggressive responses were triggered by the biomimetic shape of fish replica, or whether any intruder object was effective as well. Male fishes showed longer and higher aggressive displays as puzzled stimuli from the fish replica increased. When the fish replica emitted its full sequence of cues, the intensity of aggression exceeded even that produced by real fish opponents. Fish replica shape was necessary for conspecific opponent perception, evoking significant aggressive responses. Overall, this study highlights that the efficacy of an artificial opponent eliciting aggressive behaviour in fish can be boosted by exposure to multiple signals. Optimizing the cue combination delivered by the robotic fish replica may be helpful to predict escalating levels of aggression.

## Introduction

The use of robotics to establish biomimetic social interactions between animals and robots is a fascinating scientific field, involving both biologists and engineers. This fairly new research context investigates animal abilities, providing novel approaches to design robots with better flexible and adaptive behaviours in unstructured environments^1, 2^. On the other hand, objectivity and standardization of behaviours exhibited by robots make them advanced tools to carry out ethological investigations with a high degree of reliability^3^. Unlike classic behavioural observations, biomimetic robots, can display specific behavioural pattern “on demand”, thus enabling researchers in behavioural ecology to investigate and/or manipulate selected behaviours in animals^2–4^.

Dummies and decoys, which deliver one or more cues, have a long tradition in ethology since, in general, animals display a specific behaviour as a response to a proposed stimulus^5^, although with such strategy, weak or partial responses are obtained from subjects^6^. Compared to dummies and lures, biomimetic robots have the advantage to provide stimuli over the time and to deliver several stimuli that can be overlapped, evoking at the same time, longer and realistic interactions with animals^4, 7^. In some cases, robots and animals can affect their behaviour each other, fully closing the loop of interactions between natural and artificial agents^7–9^. Biomimetic robotic devices were successfully used to interact with several animal species. A number of studies used robots to evoke social interactions in several species, such as *Periplaneta americana* (Linnaeus)^8^, *Apis mellifera ligustica* (Spinola)^10–12^ *Gryllus bimaculatus* (De Geer)^1^, chicks, including *Gallus gallus domesticus* (Linnaeus), and *Coturnix coturnix japonica* (Temminck & Schlegel)^13, 14^, *Rattus norvegicus* (Berkenhout)^15–17^. Robotic models were used to attract individuals or small shoals in fishes such as *Danio rerio* (Hamilton)^18–25^, *Poecilia reticulata* (Peters)^9, 26^, *Gambusia affinis* (Baird & Girard)^27, 28^, *Mormyrus rume* (Valenciennes)^29, 30^, *Lucania goodei* (Jordan)^21, 31^, *Notemigonus crysoleucas* (Mitchill)^32^, and *Gasterosteus aculeatus* (Linnaeus)^33, 34^. Also, interactive robots can play a pivotal role for further progress in investigating high flexible and complex behaviours such as agonistic displays^4, 35^, however most of the studies relating to animal-robot interactions was devoted to survey group and collective behaviours^4, 8, 12, 14, 22, 23, 33^. Regarding aggressive behaviour, except for a few examples using biomimetic artefacts to evoke intraspecific aggressive response in the dart-poison frog, *Epipedobates femoralis* (Barbour), and in swamp sparrows, *Melospiza georgiana* (Latham)^35, 36^, as well as anxiety-related behaviour in zebrafish when exposed to predator-mimicking robots^37, 38^, agonistic interactions are almost unexplored by the biomimetic approach.

Aggressive behaviour is widespread across the animal kingdom because it has a key role in acquiring and defending limited resources^39–42^. Game theory predicts that evolutionarily stable strategies for conflicts occurring between conspecifics, may involve stereotyped contests featured by the ritualized exchange of agonistic cues^43, 44^. In this study, we investigated biomimetic aggressive interactions involving the Siamese fighting fish, *Betta splendens* (Regan) (Perciformes: Osphronemidae), and a conspecific-mimicking robotic fish in an open-loop agonistic interaction. Siamese fighting fishes have territorial males performing highly stereotyped and vigorous aggressive displays towards conspecific males^45–47^. On this basis, we selected this species as an animal model to explore the interactive effects of a robot inducing aggression in the aquatic environment. To trigger aggressive behaviour in *B*. *splendens* males, we developed a robotic platform including a fish replica that was inspired to the male of this species during fin spreading behaviour (an aggressive visual cue displayed by *B*. *splendens* males)^45^. Furthermore, we proposed a novel animal-robot interaction paradigm, by incorporating two red light-emitting diodes (LEDs) in the fish replica, located close to gill regions. LEDs provided luminescence as artificial surrogate of the opercular gill flaring behaviour. This behaviour is a common aggressive display in Siamese fighting fishes^45^, correlated with behavioural dominance^48^. Gill flaring display consists in the erection of gill covers, often accompanied by the dropping of the brightly red branchiostegal membranes^45, 48, 49^, thus red pigmentation has a critical role in this behaviour^49, 50^.

Several studies investigated fish behavioural responses post-exposure to artificial cues, such as pigmentations used to paint fish replicas^24, 31, 51–54^. However, no efforts focused on the effect of a light source mimicking a specific behavioural or a colour pattern of the body in animals.

The aim of this research was to use a robot to elicit aggressive responses in real Siamese fighting fishes, post-exposure to combinations of dissected cues commonly perceived by these fishes during fighting. Furthermore, we attempted to create a biomimetic fighting interaction enabling us to modulate the escalation of aggressive displays in *B*. *splendens* males. To achieve these purposes, male Siamese fighting fishes were exposed to our robotic fish replica displaying a series of stimuli singularly and combined. Indeed, different signal components can affect the receiver response depending on whether a stimulus is displayed in isolation or in chorus with other stimuli^35, 55, 56^. The highly ritualized aggressive behaviour of Siamese fighting fish includes several not physical displays (e.g. gill flaring and fin spreading) and physical acts (e.g. tail beats and bites) with which the male expresses his motivation and mate quality^47^. As a general trend in the animal kingdom, aggressive physical interaction would result in a high loss of time and energy as well as risk of injury^42, 57, 58^. Starting from these assumptions, we predicted a variation in the escalating level of aggression that characterizes the Siamese fighting fish when different animal-robot contests were presented. Particularly, Siamese fighting fish are predicted to alter the aggressive level of a fight in line with the perceived stimuli from the fish replica and this provides a way of determining whether the fish replica has been assessed as good reason (e.g. a potential opponent) to display an energy and physical costly fight to defend the territory^45, 47, 57, 58^.

In addition, the importance of fish biomimetic silhouette in evoking aggression, was also tested by replacing the fish replica with a cylindrical dummy, which should not be treated as a conspecific by real fish, contrary to what we predicted for our biomimetic fish replica. Finally, fish males responses, obtained in both these experiments, were compared with those produced in real fish-fish contexts^59^.

## Results

**Figure 1:**
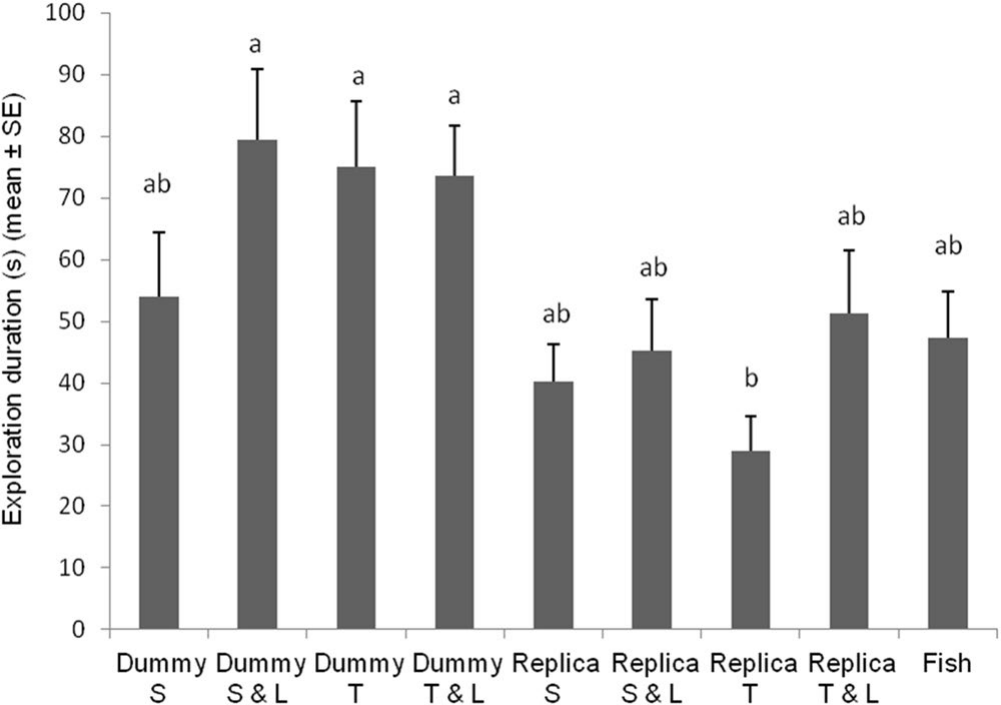
Duration of *Betta splendens* exploration post-exposure to different robot-borne combinations of fighting cues. Dummy S = cylindrical dummy static. Dummy S & L = cylindrical dummy static with LEDs on. Dummy T = cylindrical dummy twisting. Dummy T & L = cylindrical dummy twisting with LEDs on. Replica S = fish replica static. Replica S & L = fish replica static with LEDs on. Replica T = fish replica twisting. Replica T & L = fish replica twisting with LEDs on. Fish = fish vs. fish. Different letters above each bar indicated significant differences. T-bars are standard errors.

**Figure 2:**
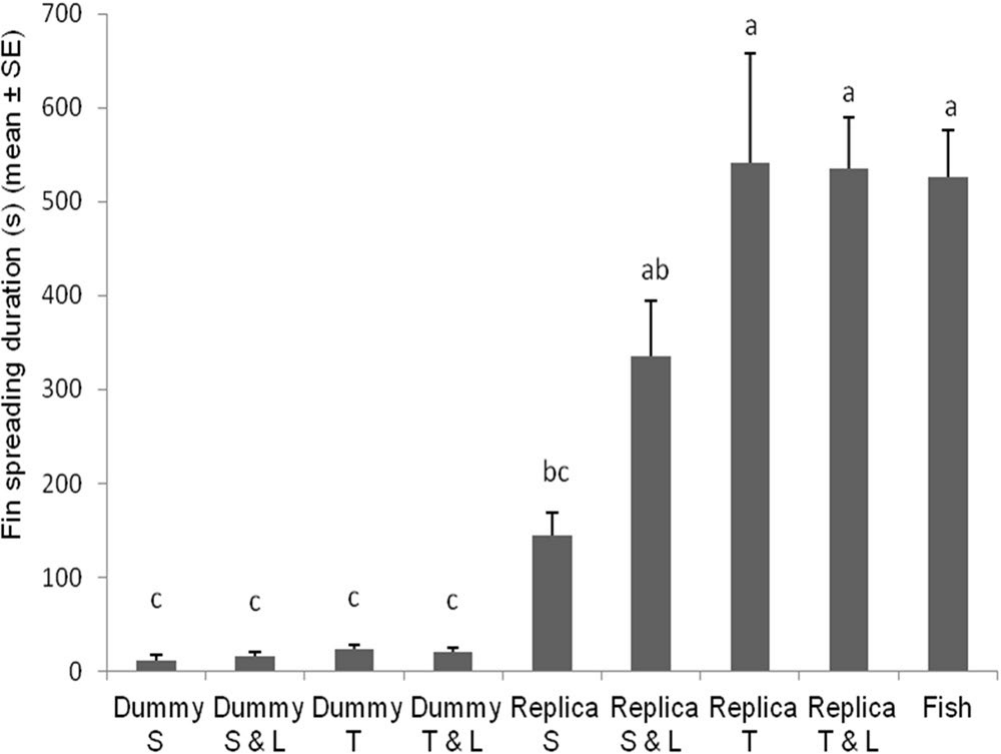
Duration of *Betta splendens* fin spreading post-exposure to different robot-borne combinations of fighting cues. Dummy S = cylindrical dummy static. Dummy S & L = cylindrical dummy static with LEDs on. Dummy T = cylindrical dummy twisting. Dummy T & L = cylindrical dummy twisting with LEDs on. Replica S = fish replica static. Replica S & L = fish replica static with LEDs on. Replica T = fish replica twisting. Replica T & L = fish replica twisting with LEDs on. Fish = fish vs. fish. Different letters above each bar indicated significant differences. T-bars are standard errors.

Our results showed that *B*. *splendens* males responded differently to various combinations of cues. Duration of exploration was significantly different in the twisting fish replica context (*F*_*8*,*112*_ = 3.8501; *P* = 0.0005). Siamese fighting fishes explored marginally longer the cylindrical dummy for contexts in which it was static with LEDs on, twisting and twisting with LEDs on (Fig. 1). Exploration lasted slightly shorter in other treatments except for the twisting fish replica context that was explored significantly shorter. Fin spreading duration was significantly affected by tested combination of cues (*F*_*8*,*112*_ = 26.2142; *P* < 0.0001). Duration of fin spreading display occurring in fish replica twisting and twisting with LEDs on contexts was comparable with the duration of fin spreading occurring during fish vs. fish context and it was longer to respect contexts in which the fish replica was static and static with LEDs on (Fig. 2). Spreading fin duration was significantly shorter when fishes were exposed to any contexts involving the cylindrical dummy.

**Figure 3:**
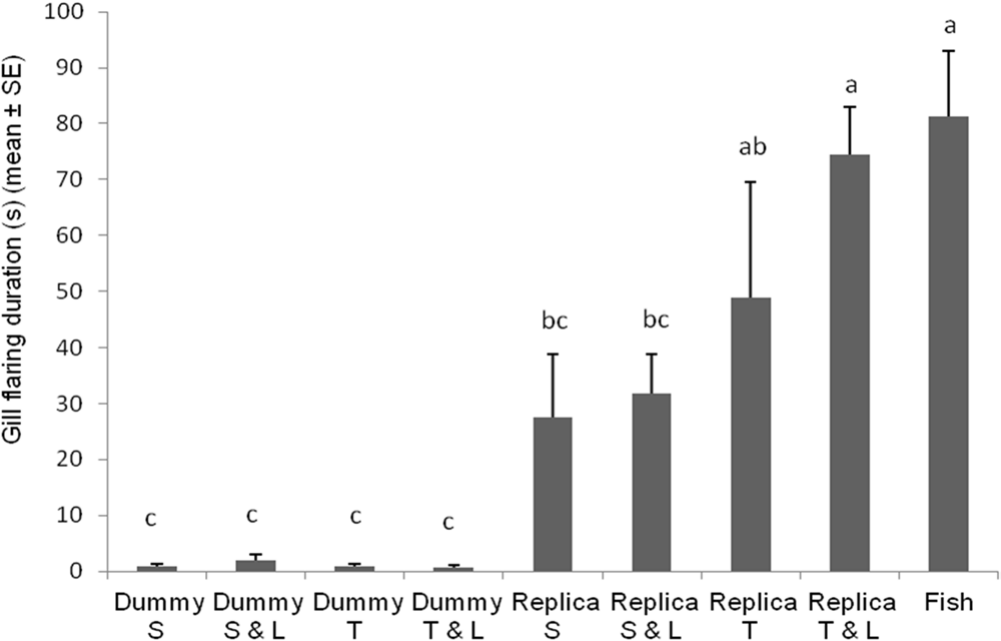
Duration of *Betta splendens* gill flaring post-exposure to different robot-borne combinations of fighting cues. Dummy S = cylindrical dummy static. Dummy S & L = cylindrical dummy static with LEDs on. Dummy T = cylindrical dummy twisting. Dummy T & L = cylindrical dummy twisting with LEDs on. Replica S = fish replica static. Replica S & L = fish replica static with LEDs on. Replica T = fish replica twisting. Replica T & L = fish replica twisting with LEDs on. Fish = fish vs. fish. Different letters above each bar indicated significant differences. T-bars are standard errors.

Gill flaring duration was significantly affected by tested combination of cues (*F*_*8*,*112*_ = 11.4135; *P* < 0.0001). Gill flaring behaviour lasted significantly longer when a fish interacted with another fish and with the fish replica which exhibited the overlap of twisting and LEDs on stimuli to respect contexts in which the fish replica just was static, static with LEDs on or twisting (Fig. 3). The duration of gill flaring display was almost nothing when we presented the four contexts replacing the fish replica with the cylindrical dummy.

**Figure 4:**
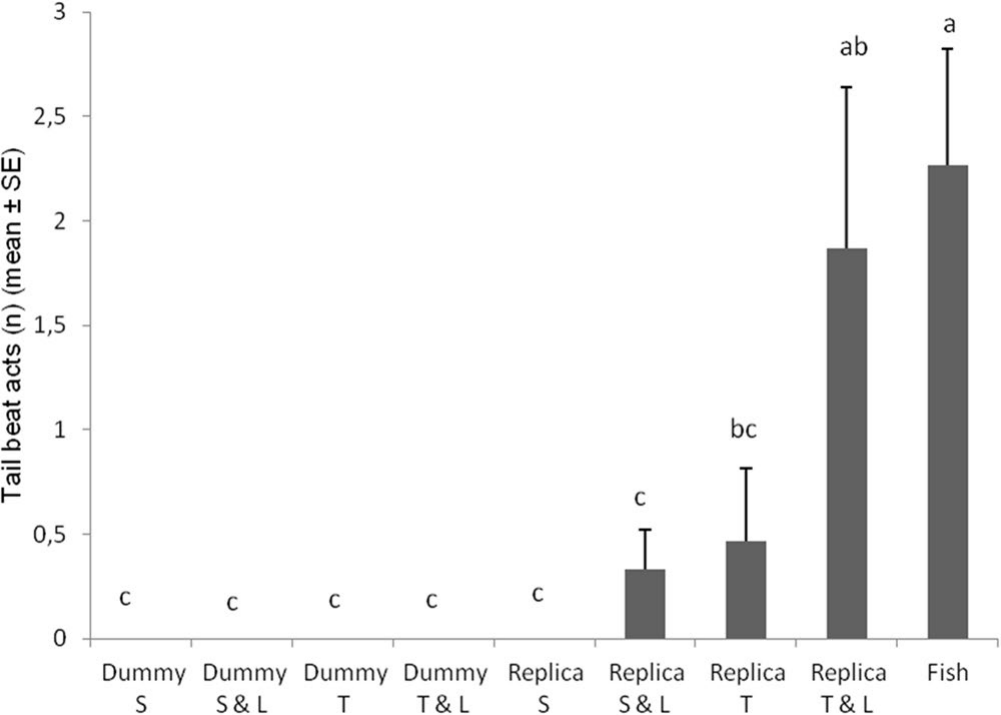
Number of *Betta splendens* tail beat acts post-exposure to different robot-borne combinations of fighting cues. Dummy S = cylindrical dummy static. Dummy S & L = cylindrical dummy static with LEDs on. Dummy T = cylindrical dummy twisting. Dummy T & L = cylindrical dummy twisting with LEDs on. Replica S = fish replica static. Replica S & L = fish replica static with LEDs on. Replica T = fish replica twisting. Replica T & L = fish replica twisting with LEDs on. Fish = fish vs fish. Different letters above each bar indicated significant differences. T-bars are standard errors.

The number of tail beat displays was significantly affected by tested combination of cues (*F*_*8*,*112*_ = 7.1096; *P* < 0.0001). Tail beat number was significantly higher when testing the fish replica twisting with LEDs on, while no significant differences were found in fish vs. fish context (Fig. 4). When fish interacted with the twisting fish replica, they exhibited a lower number of tail beats than when fish replica presented together the twisting movement with LEDs on. The number of tail beats displayed by fish towards the fish replica was lower when the replica was static or static with LEDs on, as well as towards the cylindrical dummy when it was static, static with LEDs on, twisting and twisting with LEDs on.

**Figure 5:**
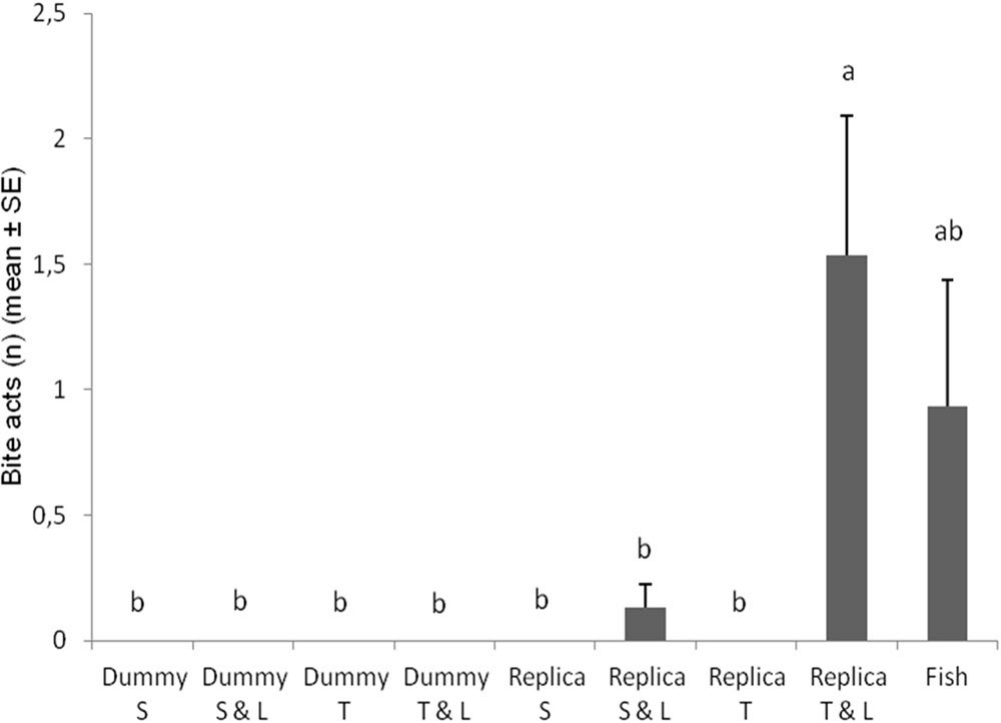
Number of *Betta splendens* bites post-exposure to different robot-borne combinations of fighting cues. Dummy S = cylindrical dummy static. Dummy S & L = cylindrical dummy static with LEDs on. Dummy T = cylindrical dummy twisting. Dummy T & L = cylindrical dummy twisting with LEDs on. Replica S = fish replica static. Replica S & L = fish replica static with LEDs on. Replica T = fish replica twisting. Replica T & L = fish replica twisting with LEDs on. Fish = fish vs fish. Different letters above each bar indicated significant differences. T-bars are standard errors.

The number of biting acts was significantly affected by the tested combination of cues (*F*_*8*,*112*_ = 4.7611; *P* < 0.0001). The number of bites towards the fish replica twisting with LEDs on was significantly higher and slightly exceed the number of bites towards real fish during fish vs. fish context (Fig. 5). Among the rest of treatments, no significant differences, regarding the number of beats, were found.

**Figure 6:**
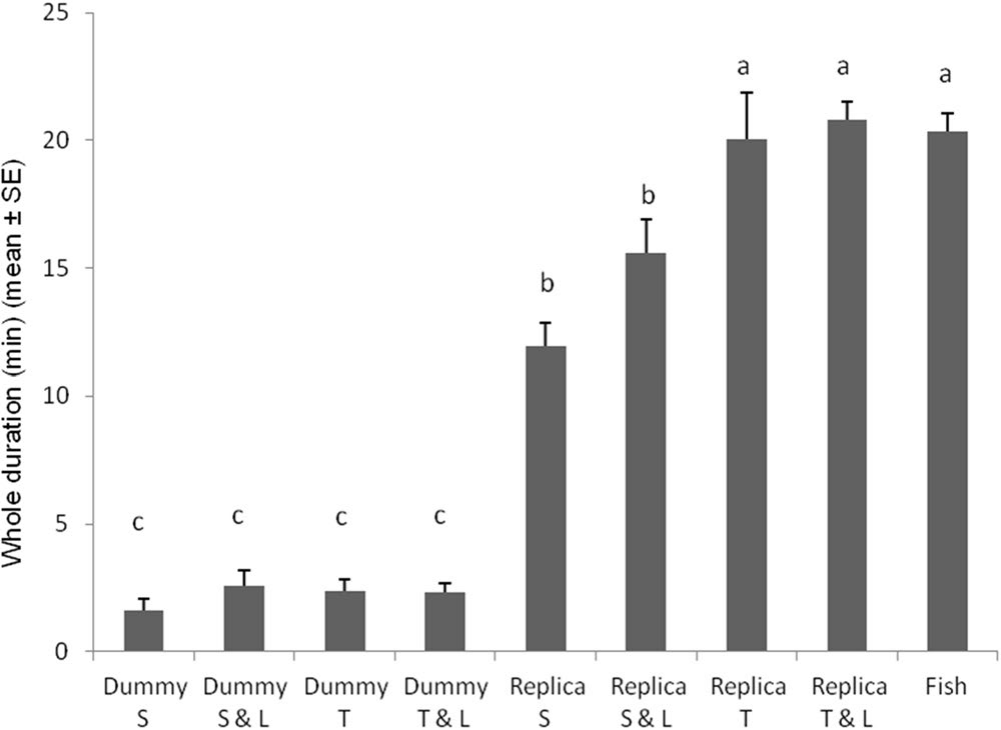
Duration of *Betta splendens* aggressive interactions post-exposure to different robot-borne combinations of fighting cues. Dummy S = cylindrical dummy static. Dummy S & L = cylindrical dummy static with LEDs on. Dummy T = cylindrical dummy twisting. Dummy T & L = cylindrical dummy twisting with LEDs on. Replica S = fish replica static. Replica S & L = fish replica static with LEDs on. Replica T = fish replica twisting. Replica T & L = fish replica twisting with LEDs on. Fish = fish vs. fish. Different letters above each bar indicated significant differences. T-bars are standard errors.

The whole aggression duration was significantly affected by the tested combination of cues (*F*_*8*,*112*_ = 89.3610; *P* < 0.0001). Duration was significantly longer when testing fish replica twisting, fish replica twisting with LEDs on and fish vs. fish. In contexts where the fish replica was static and static with LEDs on, the whole duration of the aggressive interaction was significantly lower. The whole duration of aggressive interactions was significantly lower in all the contexts where the cylindrical dummy was involved (Fig. 6).

## Discussion

The robotic approach is an elegant and innovative method to investigate animal behaviour^2–4^, with which we teased Siamese fighting fish males, triggering and modifying their aggressive behaviour. As predicted, significant differences were obtained testing different combination of cues, highlighting the communication functions that visual cues, sent by the fish replica, and how much their changes/overlapping affect specific action patterns^35, 56, 60^. In agreement with other researches^61, 62^, our observations revealed that the *B*. *splendens* male, before starting an agonistic encounter, explored the intruder agent (e.g., fish replica, cylindrical dummy, fish) for a variable period without displaying aggressive behaviours. The duration of this period depended marginally to the tested cues (Fig. 1). Particularly, the cylindrical dummy was inspected slightly longer over other treatments, but basically no main differences were found, except for the twisting fish replica. It may suggest that any foreign object in the fish territory is worth to be overseen and in the case of the twisting fish replica context, fish felt more threatened thus they preferred to exhibit aggressive displays in advance. This is also supported by the whole duration of the aggressive behaviour sequence (Fig. 6), where Siamese fighting fish males performed agonistic interactions, significantly longer towards the fish replica.

Fin spreading and gill flaring duration was longer in contexts in which the fish replica was used to respect the cylindrical dummy (Figs 2 and 3). Interestingly, a *crescendo* in duration of both displays was observed as the number of signals simultaneously emitted by the fish replica increased in the different contexts, achieving the same duration observed in the real fish context. The number of fin spreading events observed in each fish replica context was comparable with those in fish-fish encounters showing as the spread of fins was easily evoked by the biomimetic shape of the fish replica (Figure S1). Conversely, the number of gill flaring displays was affected by the fish replica different signalling. In addition, the effect of LEDs as surrogate of the gill flaring display was more appreciable in conjunction with the twist of the fish replica (Figure S2). Our data revealed that probably, the gill flaring display represents a more selective behavioural reaction over the fin spreading behaviour, probably because the ability of fish to extract oxygen from water, by ventilating their gills, is drastically limited during gill flaring^63^. Therefore, we hypothesize that gill flaring display needs a higher level of sensory information for being evoked and for increasing its performance. This showed that multiple signal systems represent an important evolved communication strategy to unlock energy costly intraspecific behavioural patterns^55, 60, 64^, adopted by this species too. Interestingly, the integration of dynamic multimodal signals was also necessary when robots were used to elicit aggression in *E*. *femoralis* and *M*. *georgiana* ^35, 36^, as well as during biomimetic predator-prey interaction in *D*. *rerio* ^37, 38^.

However, success in evoking and modulating both these displays did not guarantee the achievement of our aim in triggering aggressive behaviour. Indeed, *B*. *splendens* males perform these behaviours both in agonistic encounters, to threaten other opponent males, and in courtship interactions, to persuade females^45, 65^. Evidences of our aim achievement can be confirmed relying on triggering aggressive physical acts that *B*. *splendens* displayed towards the fish replica. In fact, aggressive behaviours are supported only if benefits of territoriality exceed costs^66^, therefore, physical combats are generally reserved to rare events in nature, in order to reduce the loss of energy, time and to avoid the risk of injuries^42, 57, 58^. Physical acts occurred when the fish replica delivered at least two stimuli simultaneously (e.g., biomimetic shape in conjunction with LEDs on) and not when the fish replica was static like a standard dummy (Figs 4 and 5). Interestingly, although the cylindrical dummy was able to turn on LEDs, twist and twist in conjunction with LEDs on, as the fish replica did, no physical aggressive displays were directed to it. Our results showed a significant lower response regarding both not physical and physical aggressive displays towards any context in which the cylindrical dummy was used. This confirms the key role played by the biomimetic shape in the acceptance of the fish replica as an opponent. Nevertheless, also the effect of realistic eyes, missing in our fish replica, is worth studying in further investigations concerning agonistic interaction, since realistic eyes led to a significant improvement of the acceptance level in a fish replica during social interactions with *P*. *reticulata* ^26^. In addition, the luminescence of LEDs as surrogate of the gill flaring behaviour and the movement of the fish replica also had a significant role, in conjunction with the shape, to modulate and increase the level of aggression in Siamese fighting fish. As further confirmation, the number of tail beats grew as the fish replica displayed more stimuli concurrently, approximating the number of acts of this behaviour displayed towards a real opponent (Fig. 4).

Remarkable were responses in terms of number of bites. Biting acts represent the culmination of the aggressive display in *B*. *splendens* males^45, 65^. Accordingly, it would be triggered by a cogent and complex communicative system. In our case, a significant number of bites was obtained when the fish replica fully displayed its stimuli concurrently (e.g., shape, LEDs on, twisting) over other fish replica and cylindrical dummy contexts, and were comparable to the number of bites displayed towards real fish (Fig. 5). Although we have to take into account that experimental conditions were slightly different in fish-fish context (e.g., a transparent partition was placed between real fish opponents to avoid injuries due to physical contacts), it can be assumed that these results are close to our predictions. However, further investigations should be carry out in future works to evaluate *B*. *splendens* responses to conspecifics by abolishing visual feedback that could affect comparisons with responses to a robotic stimulus^18, 67^. Interestingly, Ruberto *et al*.^67^ obtained an indistinguishable preference for the live stimulus and for the robotic stimulus by using one-way glass to isolate the stimulus fish that could not see the focal fish, to avoid visual feedback between the conspecifics.

The use of dummies, mirror images and video playbacks as visual cues to stimulate aggressive responses in several fish species, are widely exploited in ethological investigations, though these strategies cannot fully replace a live fish^6^. In recent years, several studies were performed in order to investigate animal social interactions trough the use of robotic replicas^8, 10–17^ and many of these studies involved fishes^9, 18–24, 29, 30, 34^. Surprisingly, few researches using robots to investigate aggressive interactions were carried out^35–38^, although aggressive behaviour represent a crucial factor in optimizing the fitness of a species^57, 58, 68^.

To the best of our knowledge, the present study represents the first attempt to use a robotic replica to investigate intraspecific aggressive behaviour in fishes and provides basic knowledge for the use of light signals mimicking specific behavioural displays during interaction with animals. Indeed, light stimuli have been used by a robot to induce a desired behaviour (e.g. collective motion), in *Artemia salina* L.^69^ since brine shrimp have phototaxis behaviour therefore they followed the light source provided by the robot. In our case, the light stimulus intended to surrogate a specific and stereotyped behaviour, displayed by the Siamese fighting fish^45, 48^, such as the gill flaring display.

Overall, we showed that our fish replica elicited aggressive behaviour in *B*. *splendens*, that escalated as the cues overlapping (e.g., shape, light, twisting), increased. The combination of stimuli emitted by the fish replica allowed us to confirm our second hypothesis, where we predicted the possibility to modulate the escalating level of aggression in *B*. *splendens* males. From an ecological point of view, our results add basic knowledge to understand key aspects of territorial aggression in Siamese fighting fishes, and may also help to develop novel reliable methods, based on a biomimetic approach, to investigate aggressive displays in aquatic animals.

## Methods

### Ethics statement

This research adheres to the guidelines for the treatment of animals in behavioural research and teaching (ASAB/ABS, 2014)^70^. All treatments of experimental animals complied with the laws of the country (Italy) in which they were performed (D.M. 116192), as well as European Union regulations^71^. All experiments are behavioural observations, and no permits are required in the country where the experiments are conducted. Before the test phase, having one animal per tank was indispensable and not considered stressful, since this is not a group-living fish but rather a high territorial species. No fishes were injured or killed during the experiments.

### Fish rearing and general observations

Male veil tail strain Siamese fighting fishes were purchased from a local aquarium store (Pontedera, Pisa, Italy). All fishes matched in size and had a blue livery, although the shade of blue was not perfectly homogeneous among subjects. Siamese fighting fishes were reared individually in tanks (28 × 14 × 15 cm), filled with dechlorinated tap water, which was completely replaced every third day. Opaque partitions were placed between tanks to avoid fishes seeing each other before the testing phase. Fishes were maintained under controlled conditions [25 ± 1 °C, 16:8 (L:D) photoperiod] at the Institute of BioRobotics (Scuola Superiore Sant’Anna, Pisa) and fed twice daily using a diet of Tetramine flake food.

Experiments were carried out from March to June 2016 in laboratory conditions (25 ± 1 °C) in a room illuminated with overhead fluorescent daylight tubes (Philips 30 W/33) [16:8 (L:D) photoperiod, lights on at 06:00]. The light intensity in close proximity of the testing arena was approximately 1000 lux, estimated over the 300–1100 nm waveband using a LI-1800 spectroradiometer (LI-COR Inc., Lincoln, NE, USA), equipped with a remote cosine receptor^40^. Directional light cues were avoided by using diffused laboratory lighting to reduce possible reflection and phototaxis.

All fishes were tested in tanks (40 × 30 × 20 cm) with their sidewalls shielded with screens of white filter paper (42 ashless, Whatman Limited, Maidstone, Kent, United Kingdom) to prevent environmental cues^41^. In each experiment, the behaviour of *B*. *splendens* was directly recorded by an observer dressed with a white coat, in order to minimize his impact^40–42, 72^. For each replicate, the test tank was carefully washed for about 30 s with warm water at 35–40 °C, then cleaned using water plus mild soap for about 5 min, rinsed with hot water for about 60 s, then rinsed with tap water at room temperature^40, 73^, and finally refilled with dechlorinated tap water at 25 ± 1 °C. Both the fish replica and the cylindrical dummy were carefully washed for about 30 s with warm water at 35–40 °C, then cleaned using water plus mild soap for about 5 min, rinsed with hot water for about 30 s, then rinsed with distilled water at room temperature, before starting each replicate.

#### Fish replica and experimental apparatus

**Figure 7:**
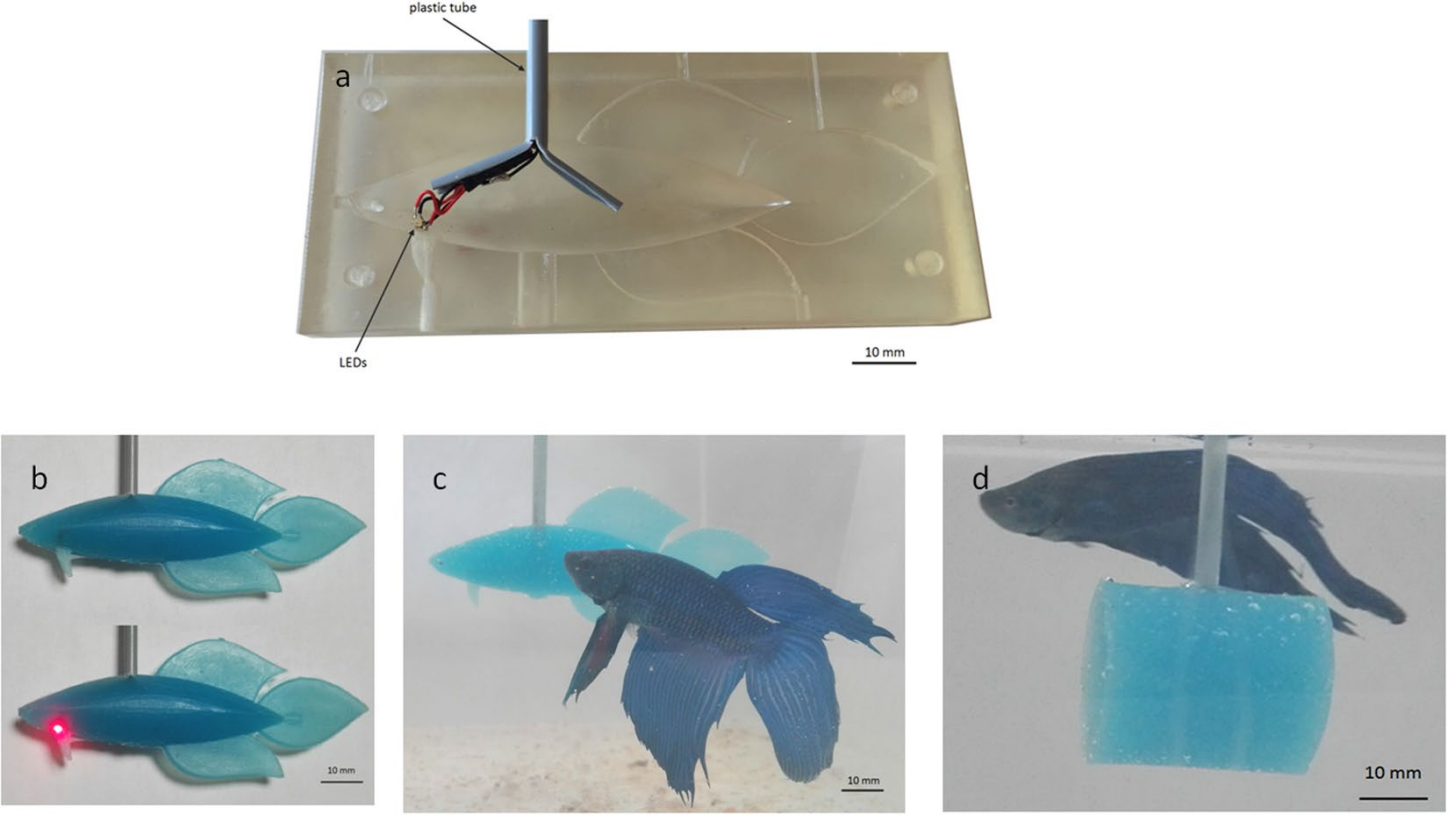
Snapshots from different *Betta splendens* fish replica development phases and the cylindrical dummy. (**a**) Right side of the replica mold in ABS during the positioning of the plastic tube and LEDs. LEDs are located close to the gill region of the fish replica shape and their wires are housed in the plastic tube. The final portion of the plastic tube was longitudinally cut and opened in order to improve its adhesion in the fish replica and facilitating the placement of LEDs. (**b**) Fish replica with LEDs off (upper) and LEDs on (lower). (**c**) Fish replica static, in the test tank, evoking the fin spreading behaviour in a *B*. *splendens* male. (**d**) Cylindrical dummy static, in the test tank with a *B*. *splendens* male.

Fish-replica design is inspired to the shape and size of *B*. *splendens* males during the fin spreading behaviour and includes fish appendages such as a dorsal fin, an anal fin, a caudal fin, two ventral fins and two ocular protuberances. Total length, height and width were 80 mm, 35 mm and 13 mm respectively. We designed the fish replica mold in SolidWorks (Dassault Systemes, Vélizy-Villacoublay, France) and printed it in a rigid acrylonitrile butadiene styrene (ABS) plastic, manufactured in rapid prototyping (Fig. 7a). Two red LEDs were positioned close to the gills region in the mold (Fig. 7a,b). Afterwards, we melded a transparent liquid silicone rubber with a nontoxic blue pigment, since all the fish we tested had blue pigmentation, and injected it in the mold until the silicon rubber dried. Fishes that we tested had variable shade of blue and generally, body coloration varies considerably in this species^74^ so we did not colour the fish replica perfectly like real fish. Concerning eyes, our fish replica had two small ocular protuberances that had the same colour of the body, although realistic coloured eyes (missing in our model), were found to significantly improve the acceptance level of a robotic fish during social interactions in some fish species^67^.

Colour measurements of the fish replica body and of incorporated LEDs (Table S1) were recorded using standard CIELab colour space coordinates determined using a spectrometer Ocean Optic HR2000-UV–VIS-NIR (Ocean Optics, USA). We believed that the body in silicone rubber of the fish replica, to respect a dummy in ABS plastic, would improve the biomimicry of the aggressive interaction as it is soft and compliant as relatively similar is the body of the fish.

**Figure 8:**
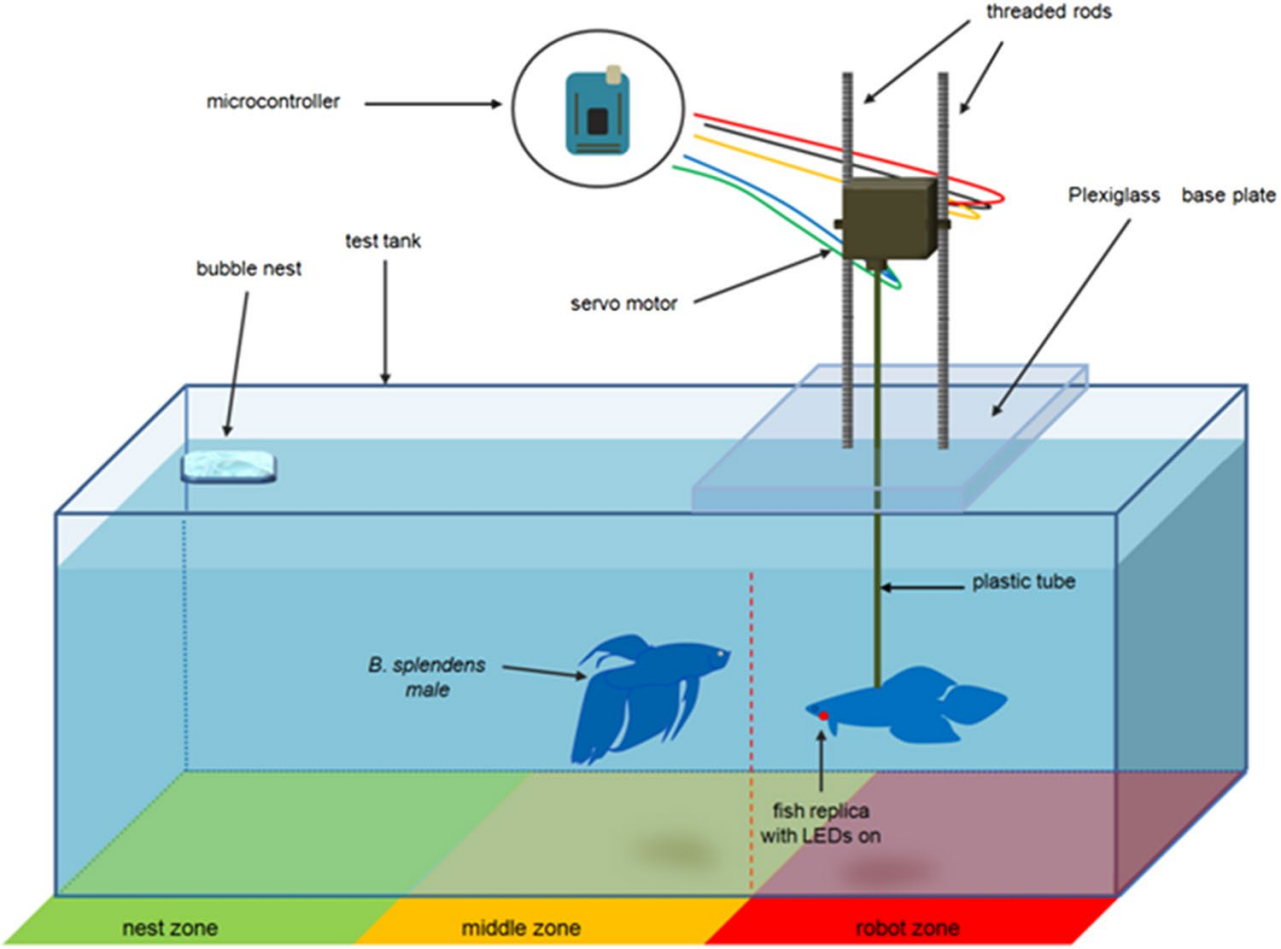
Experimental setup. The virtual division of the test tank in nest (green), middle (yellow), and robot (red) zone, is depicted below. The bubble nest is located in the nest zone. The fish replica is located in the robot zone. Tests start once an opaque partition (red dashed line), between the middle and the robot zone, is removed and the Siamese fighting fish, *Betta splendens* male can see and approach the fish replica. The fish replica is coupled to the servomotor by the plastic tube. The servomotor can be adjusted along the threaded rods to change the fish replica depth. A microcontroller was used to control both the servomotor and LEDs. The green line and the blue one, indicate wires connecting LEDs to the microcontroller. Orange, black and red line, indicate wires connecting the servomotor to the microcontroller.

The fish replica is anchored to the external apparatus by a plastic tube (length 225 mm; diameter 3,15 mm) light grey coloured, vertically inserted in the mold immediately forward the dorsal fin (Fig. 1a,b,c). The external apparatus is composed by a Robbe FS 100 Servo that was mounted on a plexiglass base plate (100 × 420 × 4,3 mm), by two threaded rods (length 180 mm; diameter 4,9 mm). This servomotor actuated the twist of the fish replica, by means of the plastic tube of the dummy (Fig. 8). Regarding the position of the fish replica in the test tank, different depths have been selected to locate fish replicas in previous studies (i.e., refs 28, 38, 46 and 67), and in some cases, depth modifications produced different responses in fish^28^. We positioned the fish replica 30 mm below the water surface^75^, approximately at the center of the robot zone of the test tank, since *B*. *splendens* guard their floating nest close to the water surface, and often, during fights, they exhibit surface breathing^45, 47, 76, 77^. Both the servo and the LEDs were controlled by an external microcontroller (Arduino Mega 2560).

### Behavioural experiments

*B*. *splendens* males were gently placed in the test tank individually at least 24 h prior performing experiments or until they build a bubble nest, since the nest presence has been found to be crucial to produce territorial males^76^. The test tank was virtually divided in three zones: nest, middle and robot zone. Test tanks were provided with a 7 × 7 cm square of bubble wrap (replaced after each replicate) on the surface of the water in one corner of the nest zone of the tank to facilitate Siamese fighting fish in building the nest and to control the nest location^76^. The fish replica was placed in the center of the robot zone of the tank (Fig. 8). An opaque partition (30 × 20 cm) prevented fish to view the stimulus until the test began and was removed after 10 min from the fish replica insertion allowing visual and physical contact. The experimental setup is depicted in Fig. 8.

15 subjects were tested and each of which interacted with the fish replica in the following contexts: (i) fish replica static; (ii) fish replica static with LEDs on; (iii) twisting fish replica; (iv) twisting fish replica with LEDs on. In the third and fourth context, the fish replica twisted of an angle of 25° and with a frequency of 0,2 Hz. As indicated by preliminary observations, these are the most suitable values to avoid suspicion and aversion in this fish as well as they are pretty similar to the body movements that we observed during not physical aggressive displays^45, 47, 48^ when a mutual assessment process occurs between two opponents in *B*. *splendens* ^45, 47, 77^. Recent studies provided evidence of the role of motor patterns on fish-robot interactions, based on that observed in live fishes^25, 67, 78, 79^.

Nevertheless, agonistic interactions are flexible and unpredictable^35, 42, 58, 80^ thus it is difficult to evaluate or reproduce the trajectory of a fish during fights. In addition, since this was the first attempt in investigating biomimetic agonistic interactions in *B*. *splendens*, we preferred to move the fish replica in a 2-dimensional regular way to have a standardized moving stimulus that does not compromise the elicitation of aggressive interactions. However, the exact role of different motor patterns needs to be further studied in *B*. *splendens* agonistic interactions.

Each context was recorded when fish started to explore the fish replica and lasted 25 min. The sequence of contexts was randomized over the experiments. Each fish was involved in subsequent experiments at least after 7 days^77^, in order to reduce any effect due to prior contexts experiences^57^, indeed the effects of context outcome appear to disappear between 24 and 48 h in Siamese fighting fishes^81, 82^.

For each context we noted: 1. duration of initial exploration (when fish noticed the fish replica and started to swim toward it and around it without performing aggressive displays); 2. duration of fin spreading display towards the fish replica, defined as the full erection of all fins^45^; 3. number of fin spreading events; 4. duration of gill flaring display towards the fish replica (the erection of gill covers, often accompanied by the dropping of the branchiostegal membranes)^45^, 5. number of gill flaring displays; 6. number of tail beats directed to the fish replica; 7. number of bites to the fish replica; 8. whole duration of the interaction, i.e. from the exploration until the end of the aggressive interaction. The behaviour of *B*. *splendens* was focally recorded by an observer^40–42, 72^.

As control, we performed two experiments: (a) fish interaction with a non-biomimetic object, and (b) fish interactions with other fishes. In order to observe if the shape of the fish replica had a relevant role to evoke aggression, the same experimental procedure was adopted to test interactions between the fish and a cylindrical dummy (length 30 mm; radius 20 mm). We positioned two red LEDs in the centre of a cylindrical mold in ABS plastic and subsequently a transparent liquid silicone rubber melded with the same nontoxic blue pigment used for the fish replica, was injected in the mold, to obtain a cylindrical dummy with the same colour and material of the fish replica (Fig. 7d).

The cylindrical dummy was mounted on the robotic platform instead of the fish replica. All the fish interacted with the cylindrical dummy in the same four contexts described above.

Concerning fish vs. fish interaction, a male was individually placed in the testing tank until he built a bubble nest. Afterwards an intruder male was inserted into the same tank, in the robot zone that was initially separated by two partitions (e.g. an opaque partition and a transparent one). After 10 min from the intruder insertion, only the opaque partition was removed, to avoid fish injuries, and the male–male interaction was observed for a period of 25 min. Overall, 9 treatments were performed: four contexts involving fish-fish replica interaction, four contexts involving fish-cylindrical dummy interaction, one context involving fish-fish interaction.

#### Data analysis

*B*. *splendens* fighting data (i.e. exploration duration, fin spreading duration, number of fin spreading acts, gill flaring duration, number of gill flaring displays, number of tail beats, number of bites, or fighting whole duration) were analyzed by JMP 10 (SAS) using the general linear mixed model (GLMM) described by Benelli *et al*.^42^. We used a GLMM with a fixed factor (i.e. the tested cue/combination of cues), which also considered ID_w_ as the w-th random effect of the individual over repeated testing phases. Averages were separated by the Tukey’s HSD test. A probability level of P < 0.05 was used to test significance of differences between means.

## Electronic supplementary material

Supplementary material

Dataset 1
