# Supplementary material for: Multiple cues produced by a robotic fish modulate aggressive behaviour in Siamese fighting fishes
Source: Sci Rep. 2017 Jul 5;7:4667. doi: 10.1038/s41598-017-04840-0 (PMC5498610; doi:10.1038/s41598-017-04840-0)
Supplement: Supplementary file 1 — Supplementary material [file 41598_2017_4840_MOESM1_ESM.doc]

**Multiple cues produced by a robotic fish modulate aggressive behaviour in Siamese fighting fishes**

Donato Romano ^1^ *, Giovanni Benelli ^1,2^ **, Elisa Donati ^1^, Damiano Remorini ^2^, Angelo Canale ^2^, Cesare Stefanini ^1,3^

^1^ The BioRobotics Institute, Scuola Superiore Sant’Anna, Viale Rinaldo Piaggio 34, 56025 Pontedera, Pisa, Italy

^2^ Department of Agriculture, Food and Environment, University of Pisa, via del Borghetto 80, 56124 Pisa, Italy

^3^ Department of Biomedical Engineering and Robotics Institute, Khalifa University PO Box 127788, Abu Dhabi, UAE

Correspondence:

* D. Romano, e-mail: address donato.romano@santannapisa.it

** G. Benelli, tel.: +390502216141, e-mail address: benelli.giovanni@gmail.com

**Supplementary material results**

The number of fin spreading acts was significantly affected by tested combination of cues (*F_8,12_*=18.5902*; P*<0.0001). The number of fin spreading events was not significantly different among fish replica contexts, and this response was comparable with the one obtained in fish vs. fish context. However, the number of fin spreading events was significantly higher in all the fish replica contexts and in fish vs. fish context to respect all the cylindrical dummy contexts (Supplementary material figure S1).

The number of gill flaring acts was significantly influenced by tested combination of cues (*F_8,112_*=20.5322; *P*<0.0001). Gill flaring acts were significantly higher in fish vs. fish context, and almost comparable with gill flaring acts performed by fish interacting with the twisting fish replica with LEDs on. The number of gill flaring acts was significantly lower in contexts in which the fish replica was static, static with LEDs on, or twisting. Also, they were lower in contexts where the cylindrical dummy was tested Supplementary material figure S2).

**Supplementary material figure S1. Number of *Betta splendens* fin spreading acts post-exposure to different robot-borne combinations of fighting cues.** Dummy S = cylindrical dummy static. Dummy S & L = cylindrical dummy static with LEDs on. Dummy T = dummy twisting. Dummy T & L = cylindrical dummy twisting with LEDs on. Replica S = fish replica static. Replica S & L = fish replica static with LEDs on. Replica T = fish replica twisting. Replica T & L = fish replica twisting with LEDs on. Fish = fish vs. fish. Different letters above each bar indicated significant differences. T-bars are standard errors.


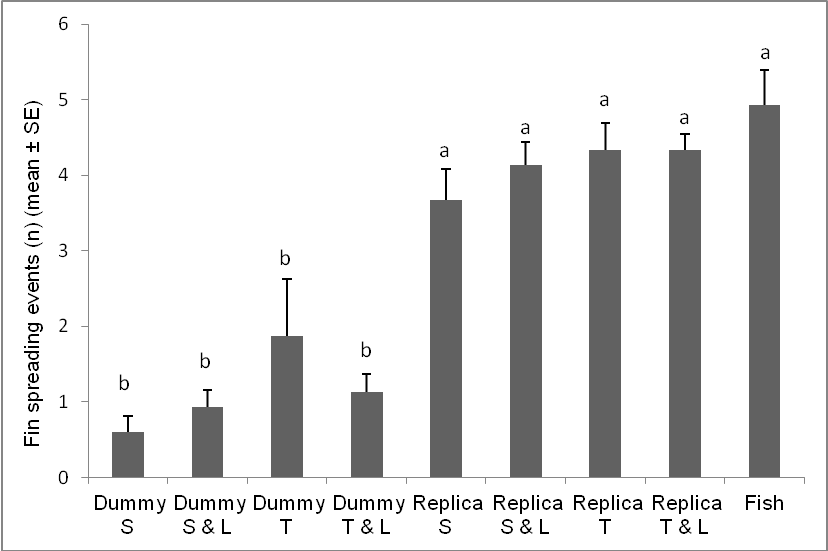


**Supplementary material figure S2. Number of *Betta splendens* gill flaring displays post-exposure to different robot-borne combinations of fighting cues.** Dummy S = cylindrical dummy static. Dummy S & L = cylindrical dummy static with LEDs on. Dummy T = dummy twisting. Dummy T & L = cylindrical dummy twisting with LEDs on. Replica S = fish replica static. Replica S & L = fish replica static with LEDs on. Replica T = fish replica twisting. Replica T & L = fish replica twisting with LEDs on. Fish = fish vs. fish. Different letters above each bar indicated significant differences. T-bars are standard errors.


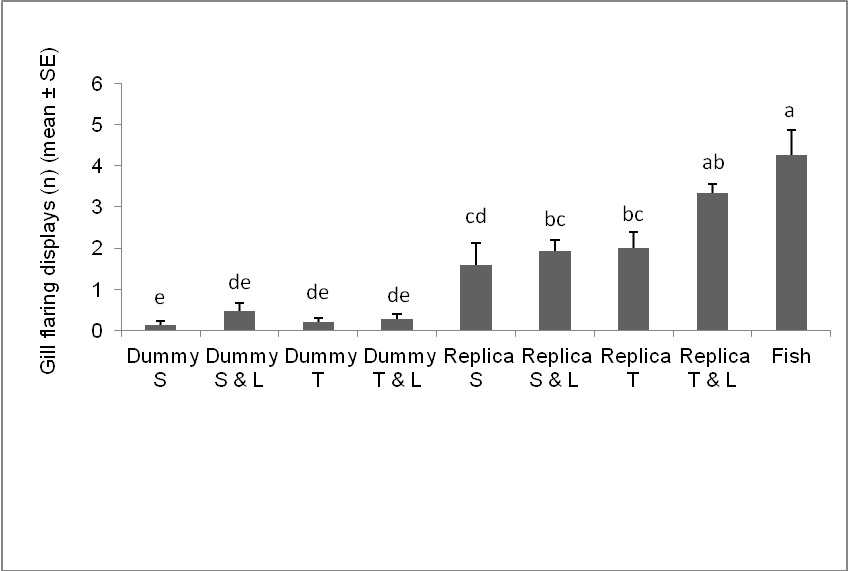


**Supplementary material table S1. Colour measurements of the fish body and incorporated LEDs ± standard error.** L* represent the lightness component, a* (from red to green) and b* (from blue to yellow) are the two chromatic components.

|  | L* | a* | b* |
| --- | --- | --- | --- |
| Fish replica body | 41.0 ± 2.31 | -7.77 ± 0.81 | -22.9 ± 2.11 |
| Red LED | 71.1 ±3.52 | 77.2 ± 3.85 | 39.5 ± 7.89 |
